# Associated Factors and Consequences of Risk of Bias in Randomized Controlled Trials of Yoga: A Systematic Review

**DOI:** 10.1371/journal.pone.0144125

**Published:** 2015-12-02

**Authors:** Holger Cramer, Jost Langhorst, Gustav Dobos, Romy Lauche

**Affiliations:** 1 Department of Internal and Integrative Medicine, Kliniken Essen-Mitte, Faculty of Medicine, University of Duisburg-Essen, Essen, Germany; 2 Australian Research Centre in Complementary and Integrative Medicine (ARCCIM), University of Technology Sydney, Sydney, Australia; University of Glasgow, UNITED KINGDOM

## Abstract

**Background:**

Bias in randomized controlled trials (RCTs) of complementary therapy interventions seems to be associated with specific factors and to potentially distort the studies’ conclusions. This systematic review assessed associated factors of risk of bias and consequences for the studies’ conclusions in RCTs of yoga as one of the most commonly used complementary therapies.

**Methods:**

Medline/PubMed, Scopus, IndMED and the Cochrane Library were searched through February 2014 for yoga RCTs. Risk of selection bias was assessed using the Cochrane tool and regressed to a) publication year; b) country of origin; c) journal type; and d) impact factor using multiple logistic regression analysis. Likewise, the authors’ conclusions were regressed to risk of bias.

**Results:**

A total of 312 RCTs were included. Impact factor ranged from 0.0 to 39.2 (median = 1.3); 60 RCT (19.2%) had a low risk of selection bias, and 252 (80.8%) had a high or unclear risk of selection bias. Only publication year and impact factor significantly predicted low risk of bias; RCTs published after 2001 (adjusted odds ratio (OR) = 12.6; 95% confidence interval (CI) = 1.7, 94.0; p<0.001) and those published in journals with impact factor (adjusted OR = 2.6; 95%CI = 1.4, 4.9; p = 0.004) were more likely to have low risk of bias. The authors’ conclusions were not associated with risk of bias.

**Conclusions:**

Risk of selection bias was generally high in RCTs of yoga; although the situation has improved since the publication of the revised CONSORT statement 2001. Pre-CONSORT RCTs and those published in journals without impact factor should be handled with increased care; although risk of bias is unlikely to distort the RCTs’ conclusions.

## Introduction

Rooted in Indian philosophy and spiritual practice, the original goal of yoga has been described as quieting one’s mind to achieve the union of mind, body and spirit [1]. Regardless of its spiritual origins, yoga has become a popular route to physical and mental well-being [1] and has been adapted for use in complementary and alternative medicine in Western society [2]. In this setting, yoga is most often associated with physical postures, breath control and meditation; and different yoga schools have emerged that put varying focus on physical and mental practices [1].

Yoga is gaining increased popularity as a therapeutic practice; with more than 20 million Americans (9% of the USA's population) reporting they practiced yoga for health reasons in 2012 [3]. From 2002 to 2012, the prevalence of yoga use increased linearly, making it one of the most commonly used complementary and alternative medicine (CAM) approaches in the US [4]. This increased use is paralleled by an increasing amount of randomized controlled trials (RCTs) of yoga; with more than 50 RCTs being published each year now [5]. Those RCTs have shown benefits of yoga for various conditions including chronic pain [6,7], cancer-related symptoms [8,9], and depression [10,11]; as a preventive means [12]; and in educational settings [13].

The methodological quality of the available evidence on complementary therapies as a whole has however been frequently questioned [14,15]. Bias in RCTs on complementary therapy interventions has been shown to depend on the studies’ origin and journal type the study is published in; with RCTs that were conducted outside the USA [16], published in CAM specialty journals [15], and/or in journals without or with low impact factor [15] being more likely to be biased towards positive results. Specifically, recent systematic reviews on yoga were frequently unable to draw definite conclusions due to the high risk of bias of the included RCTs [17–19]. Comparably to complementary therapies as a whole, it has been shown that RCTs on yoga are far more likely to be positive when they were conducted in India [20]; potentially representing an influence of location on publication bias. It has however not yet been investigated what factors might influence risk of bias in individual studies on complementary therapy interventions and whether risk of bias in individual studies distorts the studies’ conclusions towards a positive or negative interpretation of the findings. Given that a detailed analysis of all RCTs on all complementary therapies would include several thousand RCTs on very diverse interventions, this systematic aimed to assess associated factors and consequences of risk of bias in RCTs of yoga as one of the most commonly used complementary therapies [2]. A decent knowledge of underlying factors of risk of bias would help judge the applicability of yoga-based RCTs without an explicit assessment of risk of bias. Knowing the influence of risk of bias on study conclusions would be helpful when judging the expressiveness of single studies e.g. for conducting systematic reviews or designing medical guidelines. Finally, if underlying factors of risk of bias are known, caveats against those factors can be applied when designing future yoga RCTs.

The aim of this systematic review thus was to evaluate a) what factors are associated with risk of bias; and b) whether risk of bias influences conclusions in RCTs of yoga. RCTs on clinical as well as on non-clinical populations were considered.

## Methods

This systematic review is based on a previously published bibliometric analysis that descriptively summarized characteristics of RCTs of yoga [5]. Where applicable, reporting is in accordance with the Preferred Reporting Items for Systematic Reviews and Meta-Analyses guidelines [21] (S1 PRISMA Checklist).

### Eligibility criteria

#### Types of studies

RCTs, cluster-randomized trials and randomized cross-over studies were eligible. No language restrictions were applied; if necessary, language experts were consulted. Duplicate publications; this is multiple articles reporting identical or different results on already published studies; were treated as a single study.

#### Types of participants

Studies of all types of participants were eligible. No restrictions were made regarding sociodemographic characteristics or health status.

#### Types of interventions

Studies were eligible if they compared yoga interventions to one or more non-yoga interventions or untreated control groups. No restrictions were applied regarding the tradition, length, frequency or duration of the studied yoga programs. The specific yoga practices included in the intervention were not restricted as long as the intervention was based on yoga theory and/or traditional yoga practices. Studies allowing individual co-interventions were eligible while studies on multimodal interventions that include yoga amongst others were excluded. Head-to-head comparisons of different yoga interventions without a non-yoga control group were also excluded.

#### Types of outcomes

Studies with all types of outcomes were eligible.

### Literature search methods

Four electronic databases were searched from their inception through February 12, 2014: Medline/PubMed, Scopus, IndMED and the Cochrane Library. The literature search was constructed around search terms for “yoga” and a filter for retrieving randomized controlled trials [5]. The complete search strategy for Medline/PubMed is shown in Table 1. The reference lists of identified original articles or reviews and the tables of contents of the *Journal of Yoga & Physical Therapy* and the *International Scientific Yoga Journal SENSE*, which are not listed in electronic databases, were searched manually for additional eligible studies. Identified abstracts were screened independently by two review authors; and potentially eligible articles were then read in full by two review authors to determine whether they actually met the eligibility criteria.

**Table 1:** Complete Search Strategy for Medline/PubMed.

#1	Yoga[MeSH Terms]
#2	Yoga*[Title/Abstract] OR Yogic[Title/Abstract] OR Pranayam*[Title/Abstract] OR Asana*[Title/Abstract]
#3	#1 OR #2
#4	Randomized Controlled Trial[Publication Type] OR controlled clinical trial[Publication Type] OR randomized[Title/Abstract] OR placebo[Title/Abstract] OR random[Title/Abstract] OR randomly[Title/Abstract] OR trial[Title/Abstract] OR group[Title/Abstract]
#5	#3 AND #4

### Data extraction

Bibliometric data (publication year, country of origin, journal of publication) were extracted independently from the included studies by two authors using a standardized data extraction form. The journals’ 2014 impact factor was extracted from Thompson Reuters’ Journal Citation Reports [22].

The conclusions by the authors of the original articles were extracted from the studies’ abstracts and rated by a reviewer blinded to the review’s aims as a) positive: the yoga intervention was stated to be helpful for a respective condition or symptom and/or to be superior to at least one non-yoga control group; b) neutral: no clear statement regarding helpfulness or superiority regarding a respective condition or symptom was made; or c) negative: the yoga intervention was stated to be unhelpful for a respective condition or symptom and/or to be inferior or not superior to all non-yoga control groups. Where conclusions were rated to be neutral or where no conclusion was provided in the study’s abstract, conclusions from the respective study’s discussion section were additionally checked.

### Assessment of risk of bias in individual studies

Risk of bias was independently assessed by two reviewers using the Cochrane risk of bias tool [23]. Given that selection bias has been empirically shown to be the most important source of bias in RCTs [24], it was assessed as a marker of the studies’ overall risk of bias. Random sequence generation and allocation concealment were assessed as low; unclear; or high risk of bias. A low risk of bias regarding random sequence generation is assumed if an adequate random component would be included; such as random number tables, coin-tossing, or throwing a dice [23]. A high risk of bias is assumed if systematic, not strictly random, methods of allocation were used; including alteration, or allocation based on date of birth or case record number [23]. An unclear risk of bias is assumed if the information on random sequence generation were insufficient to judge the adequacy of the applied method; e.g. by simply describing the study as “randomized” without further information on the applied methods [23]. Regarding allocation concealment, a low risk of bias is assumed if proper methods were used to implement the random sequence to prevent foreknowledge of intervention assignments, including central randomization by a third party or the use of sequentially numbered, opaque, sealed envelopes which are opened sequentially [23]. A high risk of bias Is assumed if the sequence was openly accessible by the study staff [23]. An unclear risk of bias is assumed if the information on methods of allocation concealment was insufficient to judge its adequacy [23]. Discrepancies between reviewers were discussed with a third reviewer until consensus was reached.

### Statistical analysis

Data were analyzed using IBM SPSS^®^ Statistics for Windows (release 22.0. Armonk, NY: IBM Corp). Independent predictors of low risk of bias for a) random sequence generation; b) allocation concealment; and c) both domains together were identified using multiple logistic regression analysis. A backward stepwise procedure with a Wald statistic p-value of ≤0.05 with the following independent variables was used: a) publication year; b) country of origin; c) journal type of publication; and d) impact factor. Country of origin, i.e. the country where the trial was conducted, was categorized by continents as a) North America; b) Europe; c) Asia; and d) other continents. Journal of publication was categorized as a) CAM specialty journal; and b) other types of journals. Journals were categorizes as CAM specialty journals if they explicitly focused on complementary, alternative and/or integrative medicine in their aims and scopes. Journals explicitly focusing on yoga were also coded as CAM specialty journals. In a second analysis, binary predictors were used for publication year and impact factor. Publication year was categorized as a) being published before the publication of the revised CONSORT Statement 2001 [25], i.e. before 2002; and b) being published after the publication of the revised CONSORT Statement 2001; i.e. 2002 or later. Impact factor was categorized as a) journals without impact factor; and b) journals with impact factor. The second analysis was used to calculate adjusted odds ratios (OR) with 95% confidence intervals (CI) for each significant predictor. Finally, the influence of risk of bias on the articles’ conclusions was analyzed using impact factor as an independent variable and conclusion as the dependent variable. Conclusions were categorized as a) positive; or b) not positive. Given that it has previously been shown that Indian yoga RCTs are significantly more likely to reach positive conclusions than those from other countries [20], country of origin (a) India; b) other countries) was included in the analysis as a potential confounder.

## Results

### Study characteristics

**Fig 1:**
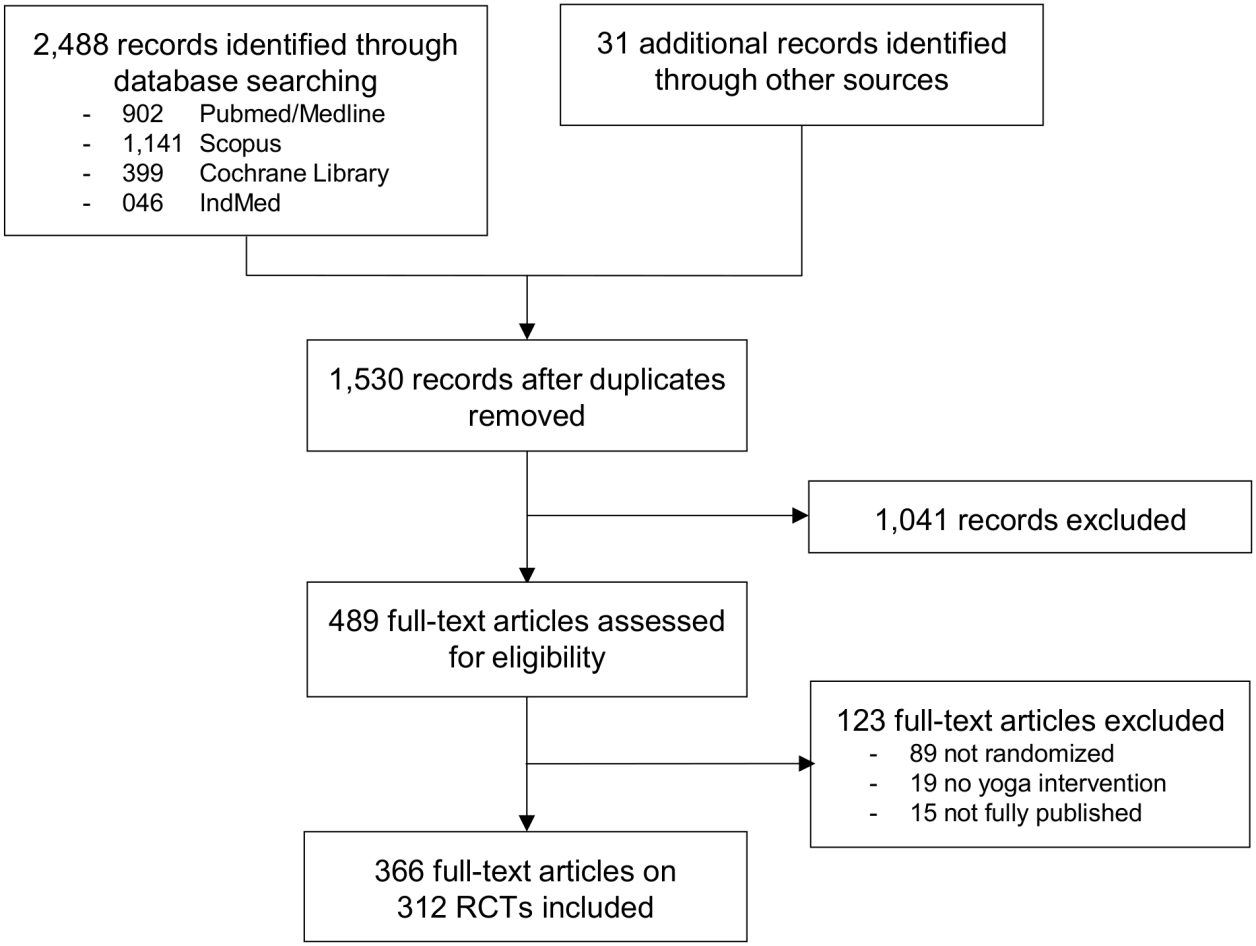
Results of the literature search.

A total of 312 RCTs were included (Fig 1). The RCTs were published between 1975 and 2014; 45 RCTs (14.4%) were published before 2001, and 267 RCTs (85.6%) in 2002 or later. Ninety-four RCTs (30.1%) originated from North-America, 37 (11.9%) from Europe, 161 (51.6%) from Asia, and 20 (6.4%) from other continents. Regarding journals, 86 RCTs (27.6%) were published in CAM specialty journals, and 226 (27.6%) in other types of journals. The journals’ impact factor ranged from 0.0 to 39.2; with a median of 1.3 (interquartile range 0.0 to 2.4); and with 131 RCTs (42.0%) being published in journals without impact factor, and 181 (58.0%) in journals with impact factor. The authors’ conclusions were positive in 278 RCTs (89.1%), neutral in 19 RCTs (6.1%), and negative in 15 RCTs (4.8%).

### Risk of bias in individual studies

Regarding random sequence generation, 120 RCTs (38.5%) had low risk of bias; 185 RCTs (59.3%) had unclear risk of bias; and 7 (2.2%) had high risk of bias. A total of 70 RCTs (22.4%) had low risk of bias; 234 RCTs (75.0%) had unclear risk of bias; and 8 (2.6%) had high risk of bias regarding allocation concealment. Combining both sources of selection bias, 60 RCT (19.2%) had a low risk of selection bias, and 252 (80.8%) had a high or unclear risk of selection bias.

### Regression analyses

**Fig 2:**
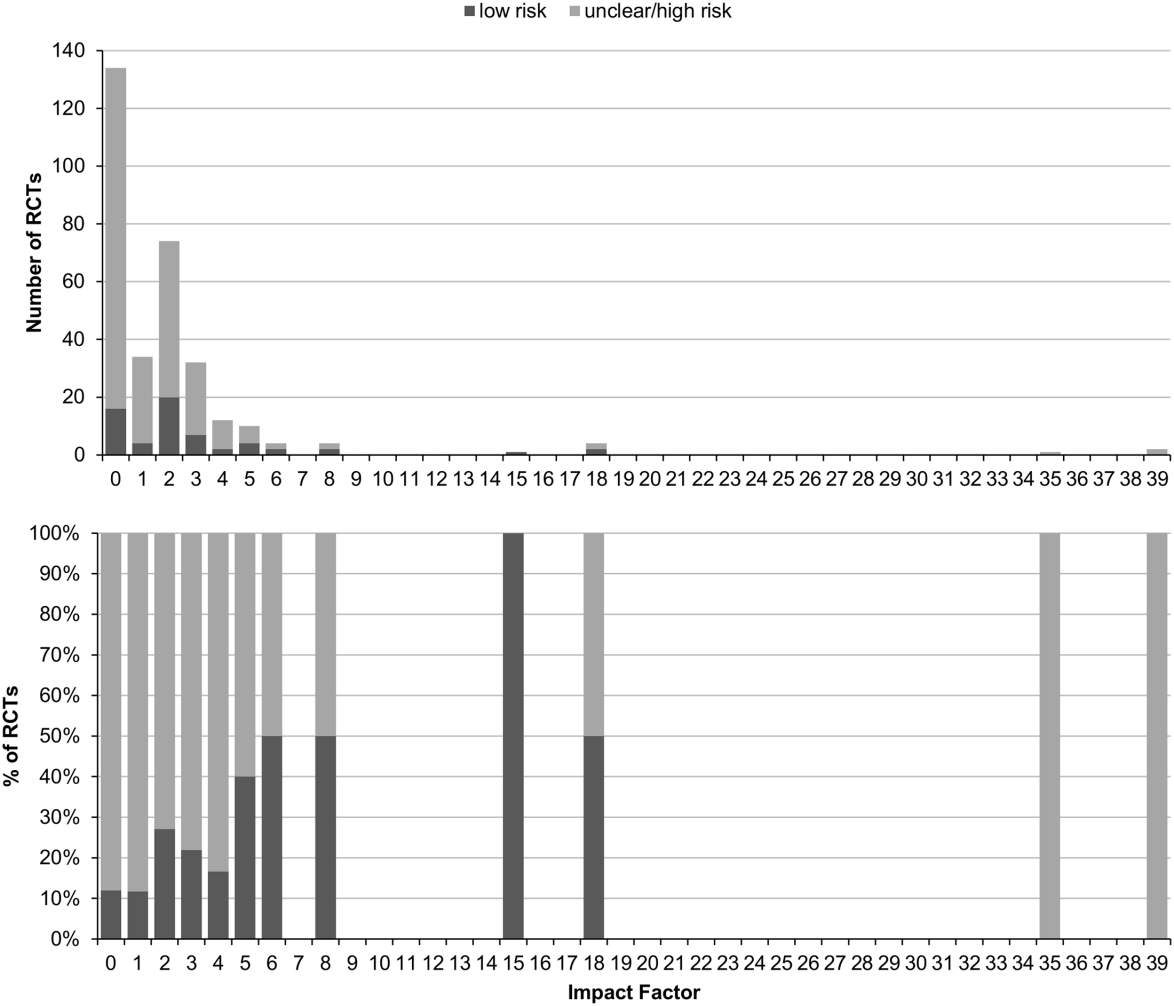
Associations of risk of selection bias and publication year in randomized controlled trials of yoga.

**Fig 3:**
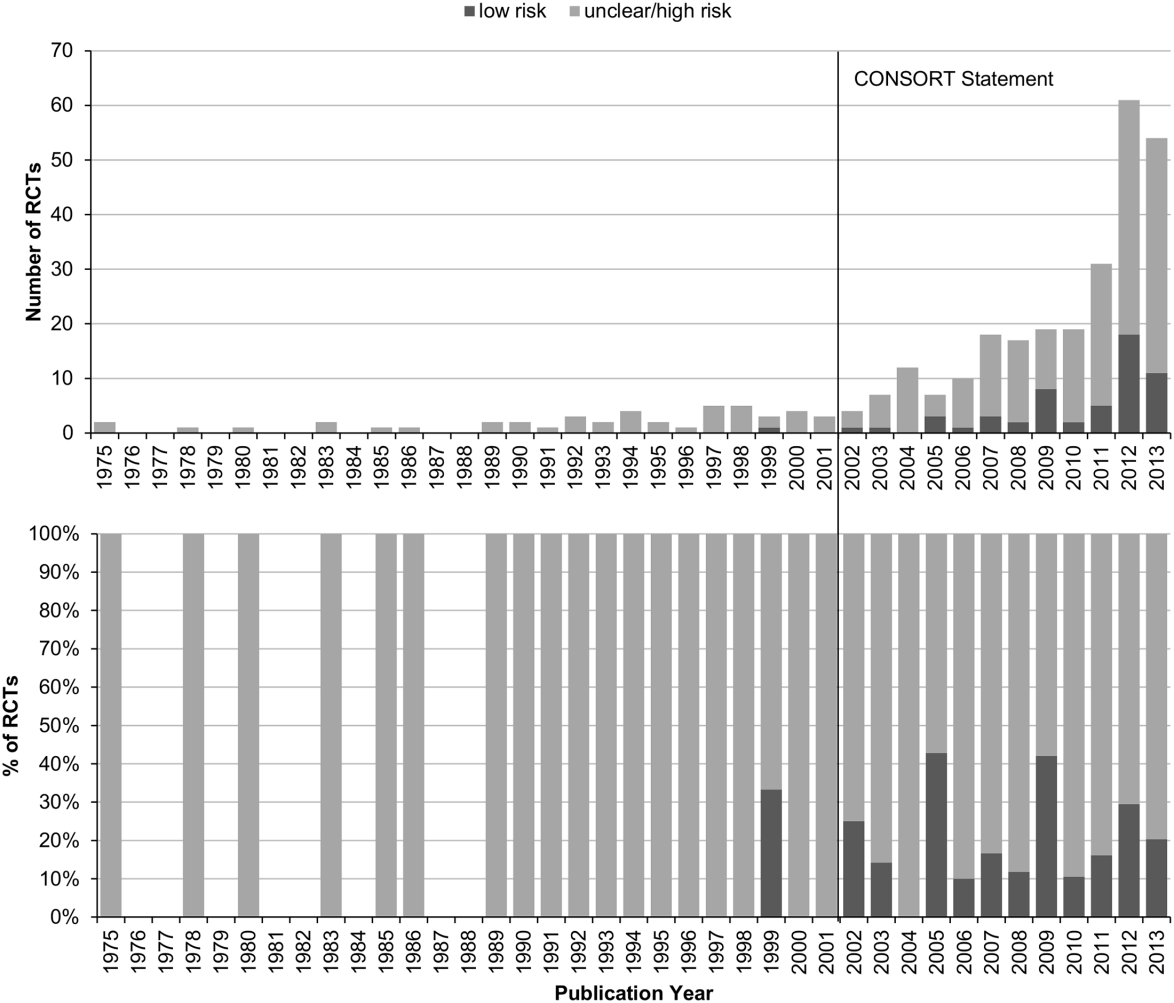
Associations of risk of selection bias and impact factor in randomized controlled trials of yoga.

Using linear predictors, only publication year and the journal’s impact factor significantly predicted low risk of bias regarding random sequence generation (p<0.001 and p = 0.015, respectively), allocation concealment (p<0.001 and p = 0.002, respectively), or both (p<0.001 and p = 0.003, respectively; Figs 2 and 3). RCTs published after the publication of the revised CONSORT Statement 2001 had 12.6 times the odds of having low risk of selection bias of those published earlier; and those published in journals with impact factor had 2.6 times the odds of those published in non-impact factor journals (Table 2). In fact, only 1 out of 45 pre-CONSORT RCTs (2.2%) had low risk of selection bias compared to 59 out of 267 post-CONSORT RCTs (22.1%; Fig 2); and 15 out of 131 RCTs from non-impact factor journals (11.5%) compared to 45 out of 181 RCTs from journals with impact factor (24.9%; Fig 3).

**Table 2:** Associations of year of publication (post-CONSORT vs. pre-CONSORT) and impact factor (with impact factor vs. without) with risk of selection bias. B, regression coefficient; CI, confidence interval; OR, odds ratio; SE, standard error.

**Dependent variable**	**Predictor variable**	**B±SE**	**P**	**Adjusted OR (95% CI)**
Low risk of bias: Random sequence generation	Post-CONSORT	2.9±0.7	<0.001	17.3 (4.1, 73.4)
	Journal with impact factor	0.8±0.3	0.002	2.2 (1.3, 3.6)
	Constant	-3.6±0.7	<0.000	-
Low risk of bias: Allocation concealment	Post-CONSORT	2.7±1.0	0.007	15.6 (2.1, 116.1)
	Journal with impact factor	0.9±0.3	0.002	2.6 (1.4, 4.7)
	Constant	-4.4±1.0	<0.000	-
Low risk of selection bias	Post-CONSORT	2.5±1.0	<0.001	12.6 (1.7, 94.0)
	Journal with impact factor	0.9±0.3	0.004	2.6 (1.4, 4.9)
	Constant	-4.4±1.0	0.001	-

Controlling for the RCT’s origin, low risk of bias regarding random sequence generation (p = 0.176), allocation concealment (p = 0.694), or both (p = 0.663) did not predict the direction of the authors’ conclusions; 225 out of 252 RCTs with unclear or high risk of selection bias (89.3%) reached positive conclusions compared to 53 out of 60 RCTs with low risk of selection bias (88.3%; Table 3).

**Table 3:** Conclusions of the included RCTs as a function of risk of selection bias.

Risk of bias	Conclusion		
	Negative (%)	Neutral (%)	Positive (%)
Random sequence generation			
High risk of bias (n = 7)	1 (14.3%)	0 (0.0%)	6 (85.7%)
Unclear risk of bias (n = 185)	7 (3.8%)	16 (8.6%)	162 (87.6%)
Low risk of bias (n = 120)	7 (5.8%)	3 (2.5%)	110 (91.7%)
Allocation concealment			
High risk of bias (n = 8)	1 (12.5%)	0 (0.0%)	7 (87.5%)
Unclear risk of bias (n = 234)	7 (3.0%)	16 (6.8%)	211 (90.2%)
Low risk of bias (n = 70)	7 (10.0%)	3 (4.3%)	60 (85.7%)
Selection bias			
Unclear or high risk of bias (n = 252)	9 (3.6%)	18 (7.1%)	225 (89.3%)
Low risk of bias (n = 60)	6 (10.0%)	1 (1.7%)	53 (88.3%)

## Discussion

### Summary of evidence

In this systematic review of 312 RCTs of yoga, only about 19% of the included trials had low risk of selection bias; and risk of bias was strongly associated with publication year. Specifically, although still highly prevalent, the percentage of trials with high or unclear risk of bias dramatically decreased after the publication of the revised CONSORT statement 2001. Further, risk of bias was negatively associated with the impact factor of the journal the respective RCT was published in; but not with country of origin and journal type. About 89% of the RCTs reached positive conclusions, however conclusions were not distorted by risk of bias.

Several findings deserve attention. Firstly the number of trials with low risk of selection bias significantly increased over time. Such increases have been found before in general medicine [25], palliative care [26] or in general medical journals as well as specialty journals [27]. The development and dissemination of reporting guidelines may be considered the main cause for these improvements of reporting of trials; with CONSORT [25,28] leading the way. However poor reporting remains common as this analysis found that only 19% of yoga trials reported adequate randomization and allocation concealment. High proportions of unclear or high risk of selection bias however are not just a problem of yoga research; they have been reported in other fields as well, for example in complementary and alternative medicine in general [14] nursing research [29], orthopedics [30] and in pediatrics [31]. Even studies published in high impact factor general medicine journals failed to reported adequate sequence generation in up to 40% of trials; and allocation concealment in up to 80% of trials from 2002 to 2003 [29]; unfortunately no recent data for those journals are available to compare the success rate in adhering to CONSORT.

Furthermore low risk of bias was associated with an existing impact factor. Previous studies have found associations between impact factor and risk of bias, with larger proportions of low risk trials being published in higher impact factor journals [32]. Of course these findings might be explained by different factors, for example trials published in higher impact factor journals differ from trials in other journals in many ways; they are more often funded, of larger size and they apply more elaborate statistics [32]. Such factors might be associated with better resources to adhere to high scientific standards of conduct and reporting. Furthermore higher impact factor journals eventually endorse the use of reporting guidelines, which in turn improves the reporting of relevant items significantly [33], even though it does not guarantee complete adherence to the guidelines [33,34]. In 2006 only 10% of journals stated that the submission of the CONSORT checklist as prerequisite for submission of manuscripts [35], and one can assume that those are probably the higher impact factor journals.

Other factors that might be taken into account when considering impact factors is the peer review process. A study revealed that one third of reviewers had not received formal training before conducting peer review of trials, even though more than 60% of them stated that the risk of bias assessment was one of their priorities when checking manuscripts [36]. Reviewers for higher impact factor journals may have more experience regarding the peer review, and the journals may even be able to provide checklist for the peer review process which are suggestive of improvement in reporting [37]. Furthermore high impact journals are often general medicine journals, and thus they may have access to a larger number of eligible reviewers. Specialty journals in general and CAM specialty journals in particular may be short of reviewers, especially reviewers with research experience and methodological training. Such journals could however provide checklists for reviewers and increase the quality of reporting of trials.

Last but not least yoga research may be special in several ways as the common guidelines may not be comprehensive enough. Studies have determined certain components of yoga interventions that should be reported in more detail [38–40]. Similar to the STRICTA statement for randomized controlled trials in acupuncture [41], a yoga addendum to the CONSORT statement may be useful to disseminate the use of reporting guidelines amongst yoga researchers.

Last but not least, results indicated that the risk of bias was not associated with the studies’ conclusions. This is in contrast with findings of associations of methodological quality of CAM trials and the likelihood of positive conclusions at least for trials published in CAM specialty journals without impact factor [15]. However, this analysis assessed study quality mainly by brief instruments such as the Jadad score rather than by more comprehensive multidimensional risk of bias tools [42]; and yoga was not assessed exclusively but only in conjunction with a variety of other CAM interventions. Thus, the present findings may indicate that other factors may be more predictive of conclusions in yoga trials than risk of selection bias. It has for example been demonstrated that Indian yoga RCTs had 25 times the odds of reaching positive conclusions than those conducted elsewhere [20]. This might be reflective of increased publication bias, inadequate statistical analysis or interpretation of findings in India; it might however also simply be due to a higher effectiveness of this indigenous practice in its country of provenance [20]. The latter interpretation would also be in line with the present findings of similar risk of bias in RCTs from different countries of origin; indicating comparable methodological quality across continents. While it has also been claimed that CAM specialty journals are more prone to positive publication bias than mainstream journals [43,44], this has not proven true for yoga RCTs whose likelihood of reaching positive results did not depend on journal type [20].

### Methodological impact

The reporting of randomized controlled trials of yoga needs to be improved. Different approaches are possible and should receive attention. Journals should ensure that they endorse the use of reporting guidelines and provide links to relevant material, publications and training resources. They might also provide checklists for reviewer, or develop templates for authors and peer reviewers enforcing a comprehensive report. Last but not least the development and dissemination of a yoga-specific addendum to CONSORT might receive special attention within the research community and foster the dissemination of CONSORT and related guidelines.

### Limitations

This analysis has a number of limitations. First, the definitions of yoga are manifold and the interventions included in this analysis ranged from highly demanding exercise-based interventions to more meditative low-impact interventions [5]. However, this is a general problem in yoga research and the exclusion of specific types of yoga might have biased the analysis toward specific time periods, countries of origin, or even single research groups. Second, no attempt was made to include unpublished trials, since the aim of this analysis included to assess the journal type, impact factor, and publication year of each included RCT. Third, only one dimension of risk of bias was assessed. As selection bias has been empirically shown to be the most important source of bias in RCTs [24], this approach seemed reasonable but might still have reduced the applicability of the findings to other sources of bias.

### Conclusion

Although the situation has improved since the publication of the revised CONSORT statement in 2001, risk of selection bias was still high in RCTs of yoga. Pre-CONSORT RCTs and those published in journals without impact factor should be handled carefully when evaluating the helpfulness of yoga for a specific patient group. However, as risk of bias is unlikely to distort the trials’ conclusions, these RCTs can still be considered if no other sources of information are available.

## Supporting Information

S1 PRISMA Checklist PRISMA checklist. (DOC)
